# Tolerogenic Dendritic Cells Derived from Donors with Natural Rubber Latex Allergy Modulate Allergen-Specific T-Cell Responses and IgE Production

**DOI:** 10.1371/journal.pone.0085930

**Published:** 2014-01-22

**Authors:** Alejandro Escobar, Adam Aguirre, María Antonieta Guzmán, Rodrigo González, Diego Catalán, Claudio Acuña-Castillo, Milton Larrondo, Mercedes López, Barbara Pesce, Jennifer Rolland, Robyn O’Hehir, Juan Carlos Aguillón

**Affiliations:** 1 Research Institute of Dental Science, Faculty of Dentistry, University of Chile, Santiago, Chile; 2 Department of Pharmacy, Faculty of Chemistry, Catholic University of Chile, Santiago, Chile; 3 Allergy Center, Clinical Hospital of University of Chile, University of Chile, Santiago, Chile; 4 Blood bank Clinical Hospital of University of Chile, University of Chile, Santiago, Chile; 5 Immunology Program, Faculty of Medicine, University of Chile Millennium Institute on Immunology and Immunotherapy, Santiago, Chile; 6 Department of Biology, Faculty of Chemistry and Biology, University of Santiago, Santiago, Chile; 7 Department of Immunology, AMREP, Monash University, Melbourne, Australia; Centre de Recherche Public de la Santé (CRP-Santé), Luxembourg

## Abstract

Natural rubber latex (NRL; *Hevea brasiliensis*) allergy is an IgE-mediated reaction to latex proteins. When latex glove exposure is the main sensitizing agent, Hev b 5 is one of the major allergens. Dendritic cells (DC), the main antigen presenting cells, modulated with pharmacological agents can restore tolerance in several experimental models, including allergy. In the current study, we aimed to generate DC with tolerogenic properties from NRL-allergic patients and evaluate their ability to modulate allergen-specific T and B cell responses. Here we show that dexamethasone-treated DC (dxDC) differentiated into a subset of DC, characterized by low expression of MHC class II, CD40, CD80, CD86 and CD83 molecules. Compared with LPS-matured DC, dxDC secreted lower IL-12 and higher IL-10 after CD40L activation, and induced lower alloantigenic T cell proliferation. We also show that dxDC pulsed with the dominant Hev b 5 T-cell epitope peptide, Hev b 5_46–65_^,^ inhibited both proliferation of Hev b 5-specific T-cell lines and the production of Hev b 5-specific IgE. Additionally, dxDC induced a subpopulation of IL-10-producing regulatory T cells that suppressed proliferation of Hev b 5-primed T cells. In conclusion, dxDC generated from NRL-allergic patients can modulate allergen-specific T-cell responses and IgE production, supporting their potential use in allergen-specific immunotherapy.

## Introduction

Natural rubber latex (NRL; *Hevea brasiliensis*) allergy is an IgE-mediated reaction to latex proteins, first described in 1979 and increasingly recognized in subsequent years [1], [2], [3]. Several NRL allergenic proteins are described, Hev b 1–13. Of these, Hev b 2, Hev b 5, Hev b 6 and Hev b 13 are the major allergens for health care workers, typically sensitized by exposure to NRL gloves [4]. Hev b 5 is a strong antigen and one of the most important latex allergens, with a high prevalence of immunoglobulin (Ig)-E reactivity in health care workers [5], [6].

Pharmacotherapy for allergic diseases, such as antihistamines or corticosteroids, ameliorates symptoms but does not stop progression; the only therapy that modifies progression of allergic diseases is allergen specific immunotherapy (SIT). However, application of this potentially curative treatment is restricted, largely due to the risk of serious adverse events, especially in asthmatics and for potent allergens such as peanut, seafood and latex [7].

Mechanisms described for regulation of the immune response to allergens include those driven by naturally occurring CD4+CD25+ Treg and inducible populations of allergen-specific, interleukin (IL)-10-secreting Type 1 regulatory T cells (Tr1) [8], [9]. Dendritic cells (DC) play a pivotal role in the immunoregulatory mechanisms underlying successful SIT, dampening allergic immune responses by absence of co-stimulation [10] or increasing IL-10 production to expand allergen-specific Tr1 cells [11], [12]. These features can be observed at an immature stage of DC differentiation [13] and in a specialized DC subset, called tolerogenic DC (t-DC) [14].

At present, allergen avoidance and symptomatic treatment for adverse reactions are the only therapeutic strategies available for NRL allergy [15]. Therefore, t-DC could provide a novel treatment strategy for inducing allergen-specific desensitization in NRL-allergic patients. Several agents have been used to generate t-DC, including IL-10 [13], transforming growth factor-β1 (TGF-β1), the active form of vitamin D3 [16], neuropeptides [17], corticosteroids and cyclosporin, amongst others [18], [19]. Glucocorticoids (GC) are potent immunosuppressive and anti-inflammatory agents used to treat autoimmune diseases and prevent graft rejection [20]. Studies have indicated that GC affects the phenotype of monocyte-derived DC, modulating their differentiation, maturation and function [21], [22].

In the current study, we aimed to generate t-DC from NRL-allergic patients using dexamethasone (dx), and determine whether dx-treated DC (dxDC) can modulate NLR-specific T-cell responses and prime T cells to become allergen-specific IL-10-producing cells. We demonstrate that dxDC have reduced alloantigen and allergen-specific T-cell stimulatory capacity and can inhibit Th2 differentiation of naïve CD4+ T cells. In addition, dxDC induced the expansion of an IL-10-producing regulatory T-cell subset with suppressive activity. These findings indicate that dxDC may be useful for antigen-specific tolerization of allergic immune responses in NRL-allergic patients.

## Materials and Methods

### Ethics Statements

The study was approved by the local medical ethics committee (Hospital Clínico Universidad de Chile Mm N° 304). All patients provided written informed consent. After the samples had been collected, each patient was allocated a trial number, demographic data collected, and the database anonymised.

### Study Subjects

Peripheral venous blood from 8 NRL-allergic patients (1–8) was collected. All patients had positive skin prick tests (≥3 mm above saline negative control) to latex extract (Stallergenes, Paris, France) and increased levels of IgE specific to an extract of NRL (Immulite 2000 analyzer; Siemens Healthcare, Germany). All patients were no atopic or sensitized to other allergens unrelated to latex. Three of eight tested patients (NRL-1, NRL-5, NRL-7) were positive for specific IgE against recombinant Hev b 5 protein, determined by ELISA. The demographic and clinical characteristics of the patients are reported in Table 1.

**Table 1:** Demographic and clinical description of study subjects.

Descriptors	Patients
Age (years)	**36.6±9.3 (range 22–54)**
Sex (male/female)	**2/6**
Main symptoms of NRL allergy	
Rhinoconjunctivitis	**1/8**
Urticaria	**8/8**
Lipoedema	**3/8**
Anaphylaxis	**2/8**
Allergy Medication	
Use of antihistamine	**7/8**
Atopy	**0/8**

All patients were defined NRL-allergic on the basis of presence of both history of allergic symptoms after allergen exposure and specific IgE and skin prick test positive to NRL.

### Preparation of T and B Cells

PBMC were obtained from peripheral blood by density gradient separation with Ficoll-Hypaque (Axis-Shield, Oslo, Norway). CD45RA+ CD4+ T cells and CD27- B cells were isolated from PBMC using antibody-coated magnetic Microbeads (MACS, MiltenyiBiotec Inc, CA, USA), according to the manufacturer’s protocol. Separation was assessed by flow cytometry (purity 90% and 97%, respectively).

### Generation of DC

DC were differentiated from monocytes using established methods [23]. Briefly PBMCs were obtained from buffy coats. Cells (3×10^7^/well) were incubated in serum-free AIM-V therapeutic medium (Gibco BLR, Paisley, UK) at 37°C, 5% CO_2_ for 2 h in a six-well plate (Falcon Becton Dickinson, Hershey, PA, USA). Non-adherent cells were removed, and the remaining cells were incubated for 7 days in the presence of 500 U/ml recombinant human IL-4 (rhIL-4) (US Biological, Swampscott, MA, USA) and 800 U/ml of GM-CSF (Shering Plough, Brinny Co., Ireland). The cultures were maintained for 7 days, replacing the medium every 2 days.

DC were left unstimulated (iDC) or matured (mDC) with 1 µg/mL of LPS from *Escherichia coli* (Sigma-Aldrich, St Louis, MO, USA) at day 6. To generate dxDC, at day 5 of the culture, dx (10^−6^ M; Sigma Aldrich) was added [24]. In some experiments dxDC were activated with 1 µg/mL LPS (LPS-dxDC). DC were routinely checked by flow-cytometric analysis (FACScanto, Beckton-Dickinson, San Diego, CA, USA) to determine expression of CD11c, HLA II (DR/DP/DQ), CD40, CD80, CD86 and CD83 (eBioscience, San Diego, CA, USA). Data analysis was performed using WinMDI 2.8 (freeware http://facs.scripps.edu/software.html).

### FITC-dextran Endocytosis Assay

Dextran uptake activity was assessed by incubating 0.5×10^6^ DC with FITC-conjugated dextran (Molecular Probes, Eugene, OR) (0.2 mg/mL) for 2 h at 37°C in the dark. Cells were washed carefully with PBS and FITC-dextran uptake was quantified by flow cytometry. Background of dextran incorporation was assessed by incubating DC on ice.

### Measurement of DC and T-cell Cytokine Production

DC (2×10^4^) were incubated with an irradiated CD40L-expressing 3T3 fibroblast cell line (cell ratio 10∶1) at 37°C and 5% CO_2_ overnight. IL-10 and IL-12 producing cells were enumerated using an ELISPOT Ready-SET-Go!® according to the manufacturer’s instructions (eBioscience). Spots were counted using A.EL.VIS ELISPOT Analysis Software (Hannover, Germany). T-cell production of IL-4 and IFN-γ was also evaluated by ELISPOT Ready-SET-Go!® (eBioscience). Tumoral necrosis factor (TNF)-α production was measured by intracellular cytokine staining and samples were analyzed by flow cytometry (FACScanto; Becton Dickinson).

### T-cell Differentiation

The Hev b 5_46–65_ peptide (TPEKEEPTAAPAEPEAPAPE), an immunodominant T-cell epitope not associated with a particular MHC II haplotype [25], was synthesized at GenScript (NJ, USA). To induce T-cell differentiation, autologous-naïve T cells were primed with 3×10^4^ Hev b 5_46–65_-pulsed DC (T_Hev b 5-DC_) (10∶1) for 6 days and rested for 4 days with 10 IU/ml IL-2 (Proleukin®, Novartis Pharmaceuticals Corporation, East Hanover, NJ, USA) in round-bottomed 96-well plates. Finally, T_Hev b 5-DC_ were harvested after 10 days and re-stimulated for 16 h with Phorbol 12-Myristate 13 Acetate (PMA)/ionomycin (Sigma-Aldrich) to assess IL-10 production by ELISPOT Ready-SET-Go!® (eBioscience) as before.

### Proliferation Assays

Allogeneic PBMC or Hev b 5-specific T-cell lines, generated using established methods [26], were labeled with CFSE (5 µM per 1×10^7^ cells) (Renovar, USA) for 15 min at 37°C. Cells were washed extensively and 2×10^5^ cells/well were cultured with Hev b 5_46–65_ peptide-pulsed DC in round-bottomed 96-well plates in serum-free AIM-V medium (Gibco BLR) for 5 days. Type II human collagen (CII)_259–263_ peptide (GIAGFKGEQGPKGET) (GenScript) was used as a control. CD4+ T-cell proliferation was determined by CFSE dilution analysis by flow cytometry (FACScanto; Becton Dickinson). Apoptosis of T cells was measured using an Annexin V Apoptosis Detection Kit APC (eBioscience).

### IgE Production

Autologous naïve B cells (1×10^5^), naïve T cells (2.5×10^5^), Hev b 5_46–65_ peptide-pulsed DC (2.5×10^4^) and CD40L-expressing fibroblasts (2.5×10^3^) were co-cultured in round-bottomed 96-well plates in the presence of rhIL-4 (1000 IU/ml) (eBioscience). After 10 days, supernatants were harvested and assessed for total and Hev b 5-specific IgE levels by Serum samples were tested for specific IgE using our standard ELISA protocol. In brief, ELISA plates (Falcon Becton Dickinson) were coated with rhev b 5 (2.5 µg/ml) [27] in 0.1 M bicarbonate buffer (pH 9.6). After blocked, diluted plasma (1/10) were added. IgE were quantified with biotinylated anti-human IgE mAb (BD Pharmingen, USA) diluted 1/1000. Development was gone with substrate solution (ATBS/H_2_O_2_). Plates were read at 460 nm using an ELISA plate reader. Background values obtained for sera and mAb on wells uncoated with Ag were subtracted from values obtained on wells coated with Ag. Values were considered positive when they differed from control supernatant values >2 times the SD.

### CD4 T-cell Suppression Assay

CFSE-labeled T_Hev b 5-mDC_ cells (3×10^5^) were boosted with mDC (3×10^4^) in the presence of increasing numbers of T_Hev b 5-dxDC_ at different ratios, in round-bottomed 96-well plates. After 7 days, T_Hev b 5_ cell proliferation was determined by CFSE dilution analysis on a FACScanto flow cytometer.

### Statistical Analysis

Results are presented as mean ± SD. The Kruskal-Wallis test with Dunn’s Multiple Comparison post-test was used to compare the mean values of cell surface marker expression, cytokine and IgE production between different cell culture conditions. Proliferative responses were compared using the Mann-Whitney test. Analyses were performed using GraphPad Prism version 5.0 for Windows, GraphPad Software (San Diego, CA, USA, www.graphpad.com). A *p* value <0.05 was considered statistically significant.

## Results

### Characterization of Tolerogenic dxDC

**Figure 1:**
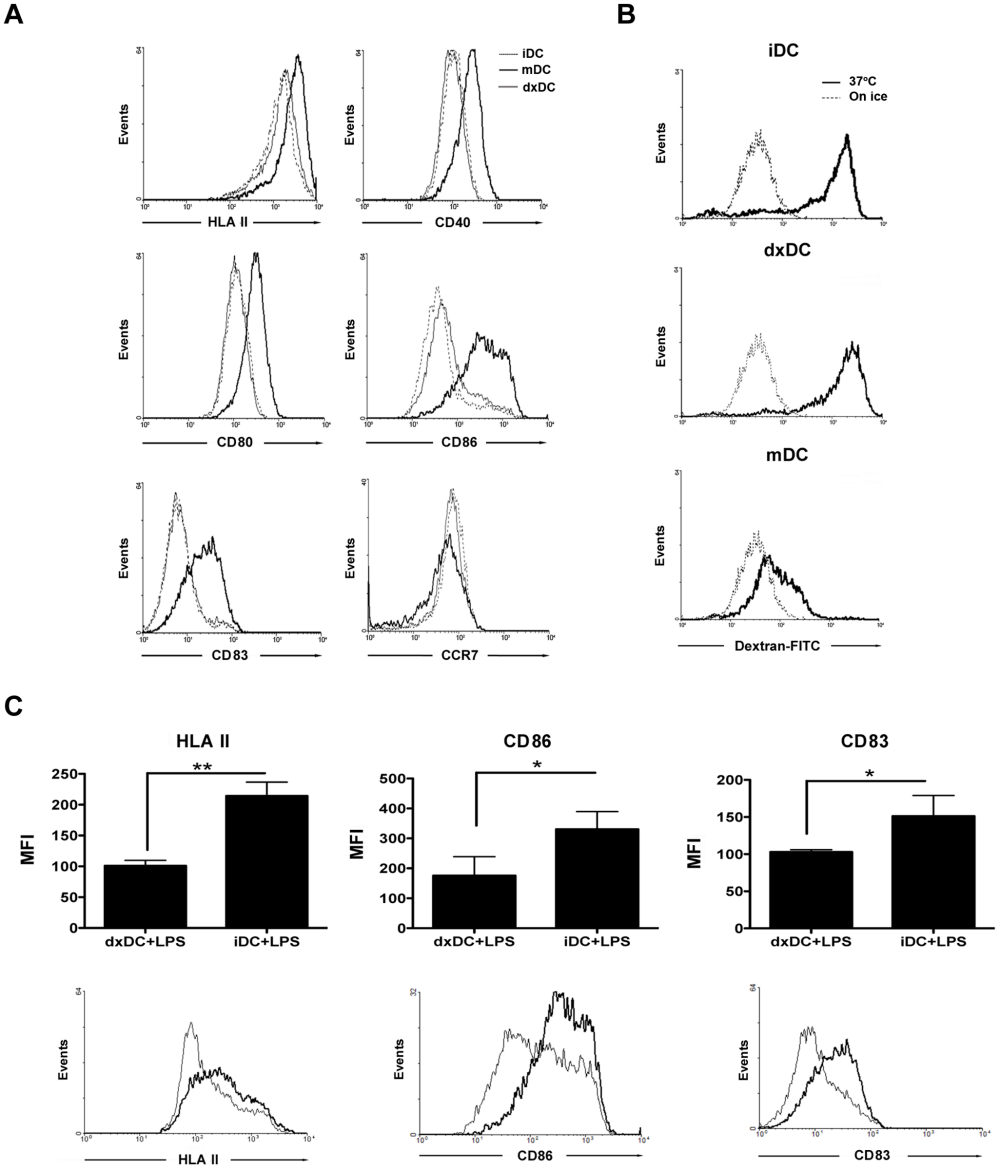
Dexamethasone-treated dendritic cells (dxDC) from natural rubber latex (NRL)-allergic patients display a stable immature phenotype. (A) Representative histograms of 1 out of 6 patients for HLA II, CD80, CD86, CD40, CD83 and CCR7 expression on immature DCs (iDC), LPS-modulated DCs (mDC) and dexamethasone-modulated DCs (dxDC). (B) Representative histograms show the uptake of FITC-dextran by iDC, dxDC and mDC. Results from 1 representative NRL-allergic patient are shown. (C) iDC and dxDC were exposed to LPS (1 ug/ml) for 18 hours. Graphs represent the mean+SD of 3 separate experiments performed in duplicate. *p<0.05; **p<0.01. Histograms show results from 1 representative NRL-allergic patient.

**Figure 2:**
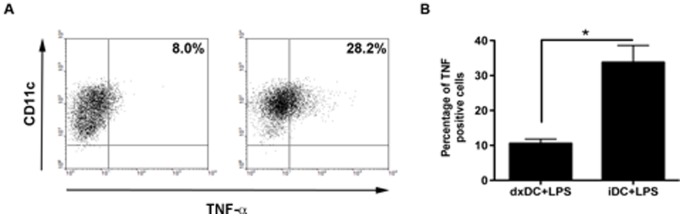
Dexamethasone-treated dendritic cells (dxDC) from natural rubber latex (NRL)-allergic patients display a stable low production of the pro-inflammatory cytokine TNF. iDC and dxDC were exposed to LPS (1 ug/ml) for 18 hours. TNF was analyzed by intracellular cytokine staining of the CD11c+ cell population. (A) Dot plots show results for LPS-stimulated dxDC (left plot) and LPS-stimulated iDC (right plot) from 1 representative NRL allergic patient. (B) The bar graph represents the mean+SD of 3 separate experiments performed in duplicate. *p<0.05.

Analysis of the phenotype and function of dxDC showed a lower level of HLA II (*p* = 0.0058), CD80 (*p = *0.0117), CD86 (*p = *0.0058), CD40 (*p = *0.0055) and CD83 (*p = *0.0058) expression compared with mDC, while the expression of the chemokine receptor CCR7 was similar to mDC (Figure 1A). In contrast, dxDC expressed equivalent levels of surface markers to untreated iDC that were used as an immature control. To further characterize dxDC, their ability to take up FITC-dextran was examined. dxDC displayed similar uptake to iDC, and higher than mDC, which showed characteristically reduced activity (Figure 1B). The suppressive effect of dx on DC was not affected by LPS, indicating that dx can induce durable immaturity of DC in terms of HLA II, CD86 and CD83 expression and production of the pivotal pro-inflammatory cytokine, TNF-α (Figure 2).

**Figure 3:**
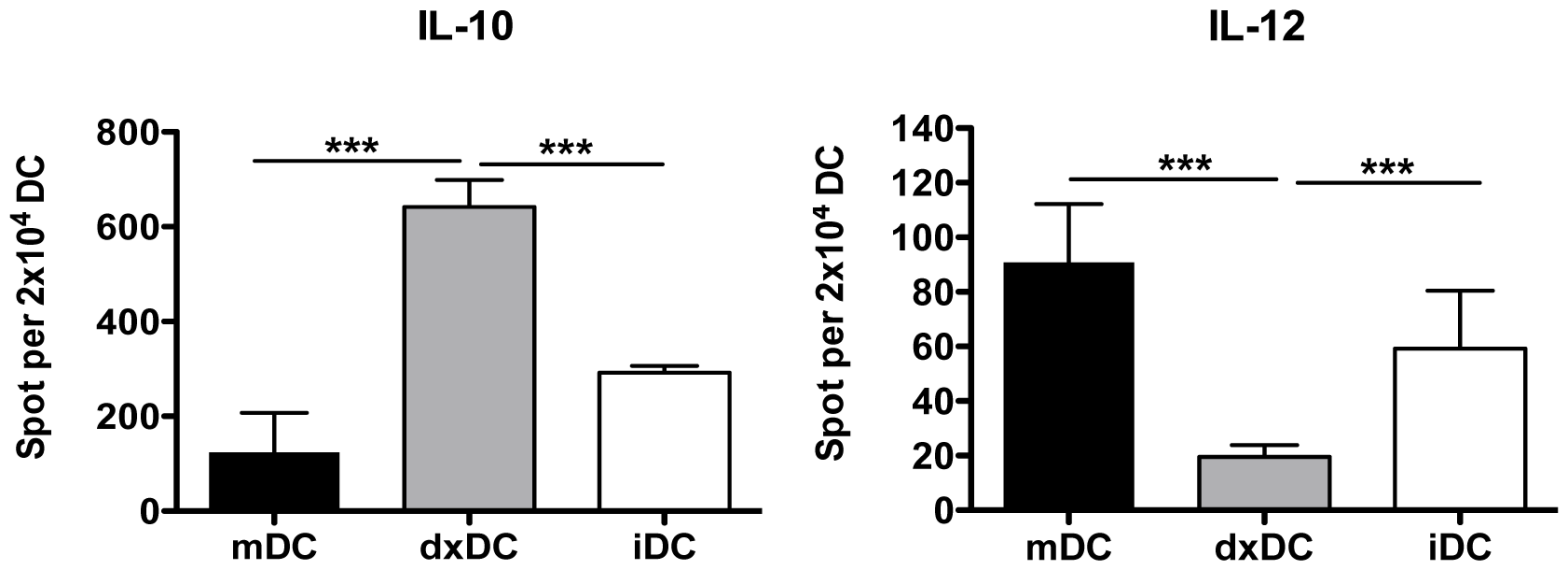
Dexamethasone-treated dendritic cells (dxDC) from natural rubber latex (NRL)-allergic patients display an anti-inflammatory cytokine profile. IL-12 and IL-10 production was analyzed by ELISPOT after DC were activated with CD40L overnight. Results are the mean+SD of 5 different patients performed in duplicate. ***p<0.001.

As cytokines secreted by DC are important in determining the differentiation fate of T cells, we assessed the cytokine profile of our DC populations. dxDC secreted high levels of IL-10 and low levels of IL-12 upon activation via CD40L. iDC and mDC produced significantly greater levels of IL-12 and lower levels of IL-10 compared with dxDC (Figure 3). These results indicate that dxDC have an anti-inflammatory profile characterized by high production of IL-10.

### Modulation of T-cell Response by Tolerogenic dxDC

**Figure 4:**
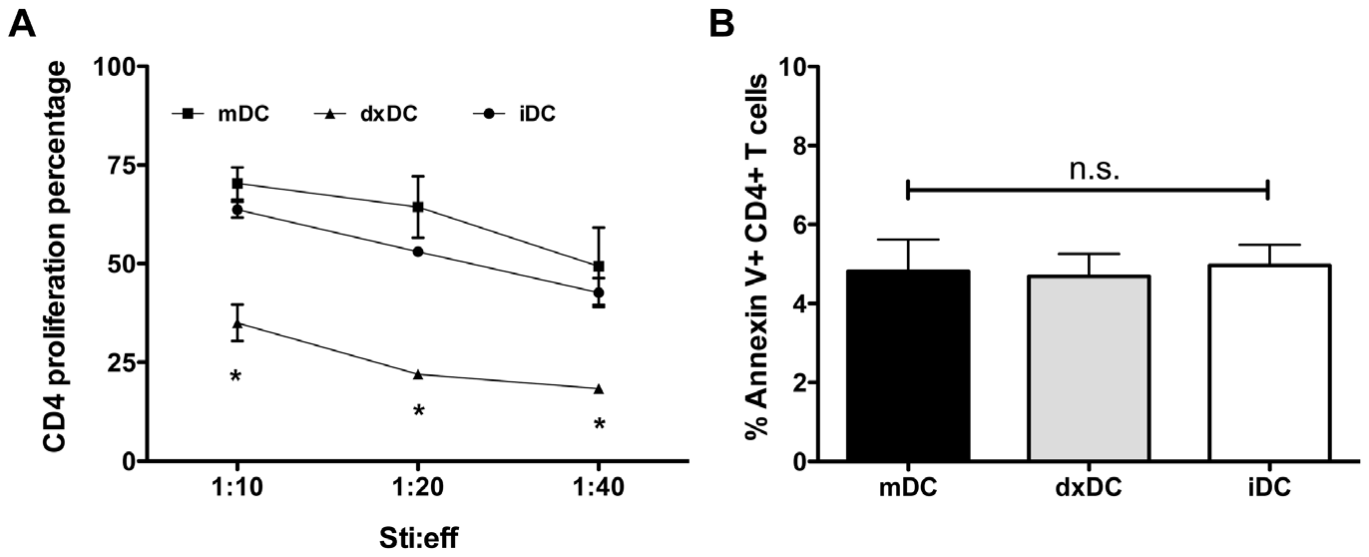
Dexamethasone-treated dendritic cells (dxDC) from natural rubber latex (NRL)-allergic patients induce alloantigen-specific T cell hyporesponsiveness. (A) Allogeneic PBMC were cultured with mDC or dxDC at different stimulator/effector ratios (1∶10; 1∶20; 1∶40). At day 5, CD4+ T cell proliferation was determined by CFSE dilution analysis. Each value is the mean ± SD of 5 different donors. (B) After allogeneic DC-PBMC co-culture, Annexin V staining was performed and the CD4+ T population was analyzed by FACS. Results are the mean+SD of 3 different donors performed in duplicate. *p<0.05; n.s. no significant difference.

Stimulation of allogeneic CD4+ T cells by dxDC at different stimulator:effector ratios induced a lower proliferative CD4+ percentage compared with allogeneic CD4+ T cells stimulated with mDC or iDC (*p*<0.05) (Figure 4A). To discard that the inhibitory effect of dxDC was mediated by a mechanism of T cell death, we examined the level of apoptosis and necrosis in CD4+ T cells by Annexin V staining and propidium iodide dye. No significant differences were observed in the fraction of apoptotic cells to T cells co-cultured with DC subjected to different treatments (Figure 4B).

**Figure 5:**
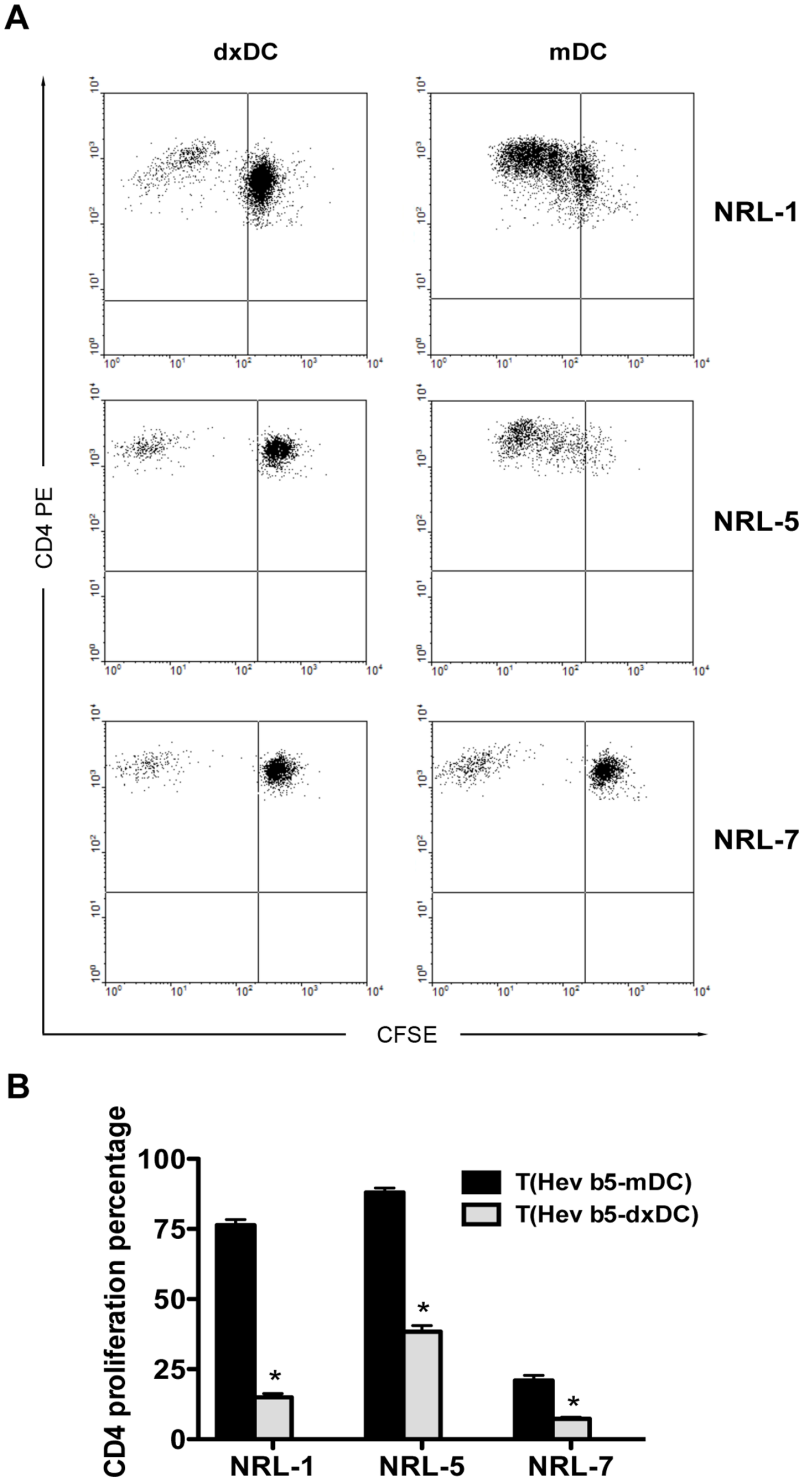
Dexamethasone-treated dendritic cells (dxDC) from natural rubber latex (NRL)-allergic patients induce allergen-specific T cell hyporesponsiveness. NRL-specific T cell lines were cultured with dxDC or mDC loaded with 10 ug/ml of NRL Hevb 5_46–64_ peptide at 1∶20 stimulator/effector ratio. At day 5, CD4+ T cell proliferation was determined by CFSE dilution analysis. (A) Representative dot plots for each of the three NRL-allergic patients. (B) The bar graph represents the mean+SD of experiments performed in triplicate on 3 patients. *p<0.05.

To determine the modulation of dxDC in NRL-specific T-cell response, oligoclonal Hev b 5-specific T-cell lines were generated from 3 patients with Hev b 5-specific IgE reactivity (patients 1, 5 and 7). These T-cell lines were stimulated with Hev b 5_46–65_-pulsed dxDC or with Hev b 5_46–65_-pulsed mDC. We observed that while peptide-pulsed mDC induced an intense proliferative response, stimulation with peptide-pulsed dxDC induced hyporesponsiveness of specific T-cell lines to their cognate antigen (*p* = 0.013) (Figure 5). T-cell lines stimulated with mDC pulsed with the irrelevant peptide CII_259–263_ did not show proliferation at all (Figure S1).

### dxDC Modulate T Cell Differentiation Hindering Subsequent B-cell Help for IgE Secretion

In order to evaluate the T-helper cytokine profile driven by dxDC, naïve CD4+ T cells from 3 patients (1, 5 and 7) were primed with autologous Hev b 5_46–65_ pulsed dxDC (T_Hev b 5-dxDC_) or mDC (T_Hev b 5-mDC_), and IL-4 and IFN-γ secreting T cells were measured (Table 2).

**Table 2:** IFN-γ and IL-4 production in primed CD4^+^ T cells.

NRL Patient N°	T_Hev b5-mDC_ IL-4/IFNγratio^1^	T_Hev b5-dxDC_ IL-4/IFNγ ratio^1^
1	30/117 = 0.26	217/295 = 0.73
5	17/132 = 0.13	160/205 = 0.78
7	4/204 = 0.01	59/313 = 0.18

Naive CD4+ (3×10^5^) cells from selected NRL-allergic patient were cocultured with Hev b 5_46–65_ pulsed mature or dexamethasone-treated DC (3×10^4^) for 6 days. Then CD4+ cells were re-stimulated for 16 h with PMA/ionomycin to assess cytokine production by ELISPOT.

1 Spots per 5×10^4^ cells.

**Figure 6:**
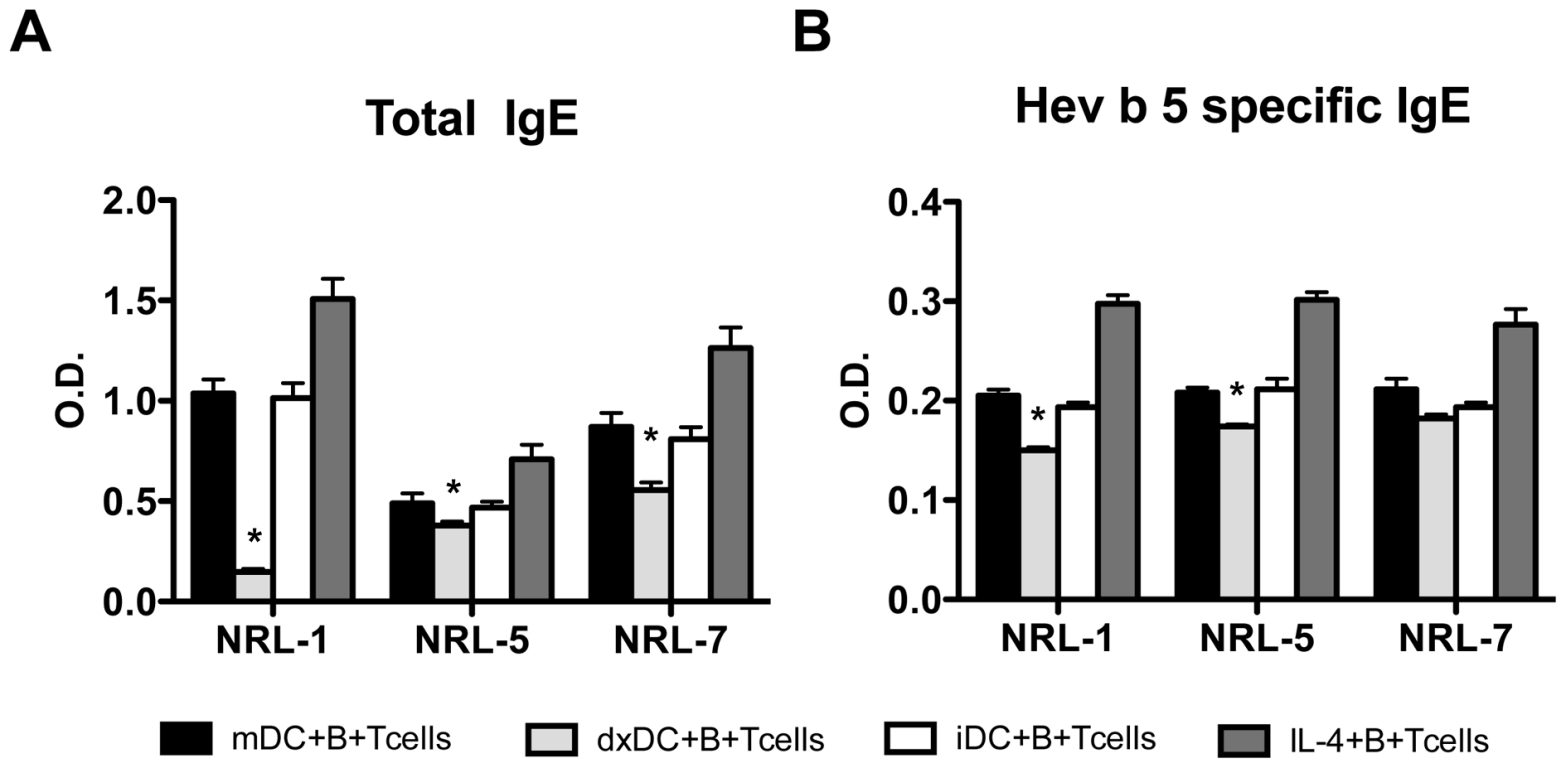
Dexamethasone-treated dendritic cells (dxDC) decrease total and allergen-specific IgE production by human autologous naïve B cells from natural rubber latex (NRL)-allergic patients. Naïve T cells and B cells were stimulated with Hev b 5-pulsed mDC or dxDC in the presence of CD40L^+^3T3 fibroblasts and IL-4 for 10 days. B cells stimulated in presence of IL-4 and CD40L were used as positive control. Total IgE (A) and Hev b 5-specific IgE (B) production was measured by ELISA. Each group of bars represents the mean+SD of experiments performed in duplicate on 3 different donors (NLR 1, 5 and 7). *p<0.05.

dxDC stimulated greater IL-4 and IFN-γ production by CD4+ T cells than mDC, but with a more pronounced effect on IL-4 production. As IL-4 favours the differentiation of naïve B cells into IgE antibody-secreting cells, we assessed the ability of T_Hev b 5-dxDC_ to induce total and allergen-specific IgE production. For this purpose, we co-cultured Hev b 5_46–65_ peptide-pulsed dxDC with autologous näive CD4+T cells, naïve B cells and CD40L-transfected fibroblasts in the presence of IL-4 for 10 days. Interestingly, total IgE (Figure 6A) and Hev b 5-specific IgE (Figure 6B) production decreased in the presence of dxDC compared to stimulation with mDC.

### dxDC Induce Regulatory T Cells

**Figure 7:**
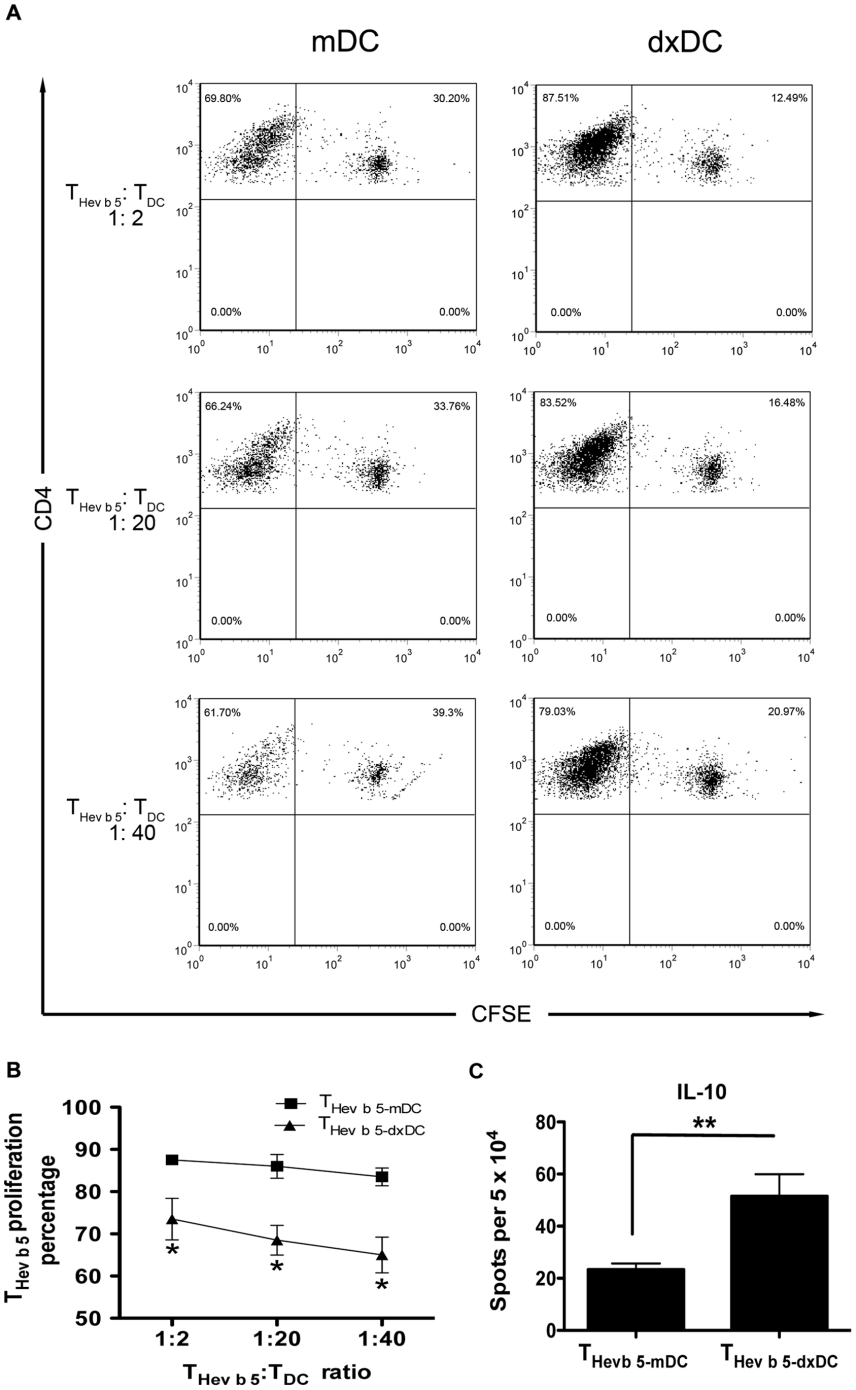
Dexamethasone-treated dendritic cell (dxDC)-primed naïve T cells (T_Hev b 5-dxDC_) exhibit a Tr1 phenotype. CFSE-labeled T_Hev b 5-mDC_ were harvested and boosted with Hev b 5 _46–65_ pulsed mDC in the presence of an increasing number of T_Hev b 5-dxDC_ or T_Hev b 5-mDC_. At day 7, T_Hev b 5-mDC_ proliferation was determined by CFSE dilution analysis of CD4+ T cells. (A) Representative dot plots. Percentages of proliferating CD4+ T cells are indicated in the upper left quadrant. (B) The graph shows the mean +/− SD of 3 different patients performed in duplicate. (C) IL-10 production by T_Hev b 5-dxDC_ and T_Hev b 5-mDC_ after PMA/ionomycin re-stimulation. The graph (mean+SD) summarizes experiments performed in duplicate on 3 different patients. *p<0.05; **p<0.01.

IL-10 secreting DC have been shown to induce Tr1 cells *in vitro* by a direct effect of this cytokine on undifferentiated CD4+ T cells [28], [29], [30]. According to these observations, we explored whether dxDC could induce the secretion of IL-10 by naïve CD4+ T cells. For this purpose, T_Hev b 5-dxDC_ were re-stimulated with PMA/ionomycin and IL-10 secretion was evaluated by ELISPOT. As expected, T_Hev b 5-dxDC_ secreted significantly more IL-10 than T_Hev b 5-mDC_ (P<0.002) (Figure 7C). To confirm that dxDC can induce a true Tr1 population, the suppressive ability of T_Hev b 5-dxDC_ was tested. Consistent with the high levels of IL-10 secretion, T_Hev b 5-dxDC_ suppressed Hev b 5-specific T-cell proliferation (Figure 7A, B).

## Discussion

iDC resident in peripheral tissues in a steady-state can induce T-cell tolerance [31]. Similarly, DC prepared *ex vivo* and exposed to antigens in the absence of full-maturation stimuli down-regulate immunity and induce tolerance. Similarly, the use of immunosuppressants such as corticosteroids can induce human DC to acquire tolerogenic properties [18], [19]. In this study, we demonstrate the feasibility of generating t-DC from NRL-allergy patients using dexamethasone. We demonstrate that dexamethasone induces an immature phenotype on DC, with low expression levels of HLA II and co-stimulatory molecules such as CD40, CD80, CD86 and CD83 and high particle uptake ability. However, the expression of the homing receptor CCR7 was not affected by dexamethasone, showing similar levels to mDC. This would enable dxDC to migrate to lymph nodes, as would be required if they were to be used in immunotherapy. Moreover, if tolerance restitution is the goal of immunotherapy, as in NRL allergy, it is essential that DC maintain an immature state in the presence of inflammatory factors. Therefore, we evaluated the resistance of the dxDC phenotype to LPS exposure. Our results demonstrate that dxDC, unlike iDC, were unaffected by LPS stimulation in terms of HLA II, CD86 and CD83 expression and production of the pivotal pro-inflammatory cytokine, TNF-α.

Low antigen presenting and co-stimulatory molecule expression levels, strongly suggest that one of the most likely mechanisms of T-cell tolerization by dxDC is through anergy induction, due to the lack of sufficient first and second activation signals [32].

Consequently, we showed that dxDC have weak allogeneic T-cell stimulatory activity. This effect was not due to deletion of alloreactive T cells, because an increase in apoptosis was ruled out. Since the aim of immunotherapy is to induce antigen-specific tolerance without impairing other immune functions, we evaluated the ability of dxDC to present the immunodominant T-cell epitope for the NRL allergen Hev b 5 [26]. Our results demonstrate that dxDC have a reduced ability to present antigen to Hev b 5 specific oligoclonal T-cell lines compared with mDC. This is consistent with the results obtained from our allogeneic experiments, and with previous data from Steinbrink *et al*. showing that clonal anergy was induced in an influenza hemagglutinin-specific CD4+ T-cell clone via stimulation with IL-10 treated DC [29].

As noted earlier, dxDC display low levels of activation signal 1 (HLA II) and 2 (co-stimulatory molecules), however, a third signal, established by secreted cytokines, is also relevant in guiding T-cell polarization [33], [34], [35]. IL-10-secreting DC have been associated with tolerance induction against common antigens [36], [37] and with poor T-cell stimulatory function [38], [39]. Furthermore, IL-10 expression has been observed in DC located in lung tissue and the intestine, suggesting an important role in maintaining local T-cell tolerance to common environmental antigens [36]. Our study shows that dxDC produce large amounts of IL-10 and low levels of IL-12 upon CD40L stimulation; these findings are in agreement with previous work using dexamethasone as a tolerogenic agent [40], [41].

As demonstrated previously, IL-10-secreting DC induce regulatory T cells upon activation [42], [43]. In line with this evidence, our study shows that naïve CD4+ T cells that were primed by dxDC (T_Hev b 5-dxDC_) differentiated into an IL-10+ T-cell population, compatible with a Tr1 cell phenotype [44]. These findings agree with those described by Bosma *et al.* for myeloid DC isolated from peripheral blood and tolerized with dexamethasone and LPS [45]. When we assessed the regulatory properties of T_Hev b5-dxDC_ cells on Hev b 5-specific-CD4+ memory T cells, we observed inhibition of T-cell proliferation, where the suppressor effect was independent of T_Hev b 5-dxDC_ cell number. This observation is compatible with an immunomodulatory mechanism of T-cell response, mediated by cytokines such as IL-10 [46], however, we do not exclude regulatory activity by other molecules. The determination of the full range of cytokines and/or molecules involved in the suppressor activity of T_Hev b 5-dx-DC_ needs further investigation.

In this study, we analyzed the influence of dxDC on T cell help for total and allergen-specific IgE production by autologous B cells from NRL-allergic patients. We observed that the presence of dxDC inhibits the secretion of total and Hev b 5-specific IgE by B cells. The reduction of IgE production could be explained by the induction of a Tr1 population. Kanjarawi *et al*. reported that CD4+CD25+ regulatory T cells were able to modulate total and β-lactoglobulin-specific serum IgE production in a food allergy murine model [47]. IL-10 appears to be an important cytokine in the success of specific immunotherapy in allergy since it has been described to be increased in blood and affected tissues of treated patients [48], [49], [50]. Akdis *et al.* reported that Tr1 cells consistently represent the dominant T cell subset specific for common environmental allergens in healthy individuals [8]. They suggested that Tr1 cells might control allergen-specific Th2 responses in allergic and healthy individuals. Pacciani *et al*. demonstrated that IL-10-producing DC can induce specific T cells with suppressive activity on allergen-specific Th2 cells from house dust mite-allergic patients [30]. In addition, experiments in murine models showed that DC transduced with IL-10 negatively regulate allergic inflammation of airways by inducing IL-10-producing T cells [51]. T cells transduced to express IL-10 have also been shown to inhibit airway hyper-responsiveness induced by Th2 cells [52]. Taken together, these studies suggest that the DC-induced deviation in T cell cytokine production towards IL-10 could be an important factor in preventing allergic responses.

In summary, our results demonstrate that Hev b 5-pulsed dxDC-primed näive T cells become Tr1 cells, which modulate allergen-specific T-cell responses. These findings indicate that dxDC may be a useful immunotherapy tool for antigen-specific down-regulation of allergic immune responses in NRL-allergic patients.

## Supporting Information

Figure S1 **T-cell lines stimulated with mDC pulsed with the irrelevant peptide CII_259–263_.** NRL-specific T cell lines were cultured with mDC loaded with 10 ug/ml of NRL Hev b 5_46–64_ or CII_259–263_ peptide at 1∶20 stimulator/effector ratio. At day 5, CD4+ T cell proliferation was determined by CFSE dilution analysis. (A) Representative dot plots for stimulation with Hev b 5_46–64_ (left), CII_259–263_ (centre) and no antigen (right). (B) The bar graph represents the mean+SD of experiments performed in triplicate on 3 patients.
(TIFF)
